# Evaluating the Effectiveness of Video Laryngoscopy Versus Direct Laryngoscopy as a Training Tool for Pediatric Intubation Skills in Simulation Settings

**DOI:** 10.7759/cureus.102658

**Published:** 2026-01-30

**Authors:** Natalie H Markley, Tori Knapp, Elias Bouyounes, Saif Ghanayem, David Redden, Hanna Sahhar

**Affiliations:** 1 Pediatrics, Edward Via College of Osteopathic Medicine, Spartanburg, USA; 2 Center for Simulation and Technology, Edward Via College of Osteopathic Medicine, Spartanburg, USA; 3 Research and Biostatistics, Edward Via College of Osteopathic Medicine, Auburn, USA; 4 Pediatric Intensive Care Unit, Spartanburg Regional Healthcare System, Spartanburg, USA

**Keywords:** direct laryngoscopy, endotracheal intubation, medical students education, pediatric intubation, video laryngoscopy

## Abstract

Introduction: Training U.S. accredited medical school students in pediatric endotracheal intubation is currently underdeveloped, with significant room for improvement. While research has consistently demonstrated the advantages of video laryngoscopes (VL) over direct laryngoscopes (DL) in adults, there is limited data on the effectiveness of VL in training for pediatric simulation. Neonatal intubation presents a high rate of adverse events. Additionally, success rates for neonatal intubation are notably low among junior residents. The integration of simulated intubation training during U.S. medical students' first two years of training remains limited and poorly researched.

Study question: This study evaluated and compared the effectiveness of VL versus DL as training tools for medical students performing intubation on pediatric patients. Which method better enhances intubation success rates and improves technical skills during the training process?

Methods: Ninety-three medical students in their second year of training out of four with no or minimal prior experience in intubation were randomized into two groups, Group A and Group B. The groups differed in the sequence of intubation equipment use. All participants were exposed to a tutorial video before intubating. Number of attempts and time to successful intubation were recorded.

Results: Tests of carry-over effects were not significant for time to intubation, number of attempts, and proportion failing. We observed that VL produced a statistically significant lower intubation time among all participants.

Conclusion: Insufficient training in pediatric intubation, such as identifying anatomical landmarks and mastering correct techniques, may contribute to the high rate of adverse effects in real-life intubation rates. This study shows the importance of providing medical students with accurate, hands-on training in pediatric intubation. The VL has the potential to enhance the students’ understanding of key anatomical landmarks and proper intubation techniques. Further research is needed to fully support or reject the hypothesis that teaching with VL will improve intubation success rates.

## Introduction

Training medical students in pediatric endotracheal intubation is currently underdeveloped, with significant room for improvement. Despite advancements in simulation technology and training methods in residency programs, the integration of these techniques during medical students’ didactic years remains limited. Given the increased difficulty of pediatric intubation compared to adults, incorporating simulation-based intubation training with video laryngoscopes (VL) during didactic years is essential. While research has consistently demonstrated the advantages of VL over direct laryngoscopes (DL) in adults, there is limited data on the effectiveness of VL in pediatric simulation training for medical school students. One study found that neonatal intubation success rates were low among junior trainees, prompting the development of a standardized training package [1]. Following implementation of the program, which included video laryngoscopy, preprocedure pauses, and standardized instruction, first-attempt success rates improved significantly, rising from 37% to 61%. Overall, the training package led to a notable increase in intubation success across all trainee levels [1]. These statistics underscore the urgent need for enhanced training methods across multiple pediatric populations.

Another study demonstrated a 23% improvement in resident performance after training and a 77.8% first-attempt success rate in real pediatric emergencies, underscoring the value of early, mastery-based skill development before clinical practice [2]. These findings highlight the need to incorporate robust simulation training early in medical education to close skill gaps and enhance patient outcomes. Early mastery of intubation during the didactic years may ultimately improve first-attempt success rates and reduce the risk of complications. This is particularly important given that unsuccessful intubations in pediatric patients are associated with higher rates of neurological injury, cardiac arrest, and mortality [3].

The objective of this study is to determine whether VL reduces intubation time and number of attempts when compared to DL in medical students performing endotracheal intubation on simulated pediatric manikins. The findings will guide recommendations on the most effective intubation training tool for medical schools. Preliminary findings from this study were presented as a poster at the Edward Via College of Osteopathic Medicine (VCOM)-Carolinas Research Day, Spartanburg, South Carolina, in February 2025 and at the American College of Osteopathic Pediatricians Conference, Alexandria, Virginia, in April 2025.

## Materials and methods

A comparative study involving 93 second-year medical students was conducted at the simulation center on the Carolinas campus of VCOM in Spartanburg, South Carolina, to assess time to successful intubation and number of attempts based on sequence of laryngoscope equipment use. VCOM institutional review board approval was obtained prior to beginning this study to confirm compliance with ethical standards and protection of participants’ rights (approval 2024-209). This study was conducted over the span of three weeks. A recruitment email was sent to all second-year medical students except those conducting the study. Each individual reviewed the study’s purpose, procedure, duration, risks, benefits, confidentiality, and all other information with ample time given to address any questions they may have in person. After completing the consent form, participants responded to a pre-questionnaire assessing their intubation experience and skill level, such as novice, intermediate, and master (Appendix 1). Participants selected novice if they had practiced on a manikin during a training event, intermediate if they had one to three years of experience in the field where they performed the skill occasionally, and master if they had more than three years of experience in the field where they performed the skill regularly. Before conducting the simulation, all participants were provided an instructional video created by faculty at VCOM-Carolinas demonstrating how to intubate using the DL or VL

The video illustrating the VL technique was one minute and 18 seconds long, and included visual representation of a physician using their left hand to grasp the ProVu blade (Flexicare Group Limited, Irvine, CA, USA), opening the manikin’s mouth with the cross-finger technique, inserting the blade into the midline of the oral cavity, avoiding sweeping of the tongue and placing the blade down the midline until the base of the tongue was reached. The physician then showed how to apply pressure caudally and upward with the handle at a 45 degree angle to the bed if necessary to elevate the epiglottis and observe the vocal cords. The video then showed what would be seen on the monitor when visualizing the vocal cords. Next, instructions were given to grasp the endotracheal tube with the stylet inserted in the right hand, to advance the tube into the right side of the mouth and to advance it under direct supervision until the base of the tongue. The video then instructed to turn the attention back to the monitor and advance the endotracheal tube through the vocal cords until the cuff is no longer visible. Instructions were given to firmly hold the endotracheal tube in place, withdraw the blade, remove the stylet, connect the Ambu bag to the endotracheal tube adapter, and give three breaths and watch the lungs inflate. The video stated that seeing the lungs inflate is confirmation that the endotracheal tube is in the trachea, and not the esophagus. The video illustrating the DL technique was one minute and 16 seconds long, and included the same information as stated above; however, the physician used a Mac blade and did not instruct viewers to use a monitor during the technique.

Simulations were conducted individually with participants intubating a pediatric intubation trainer manikin equivalent to a six-year-old pediatric patient. The study used the AirSim® Child X from Trucorp (Lurgan, UK). The participants were split into two groups: Group A performed intubation using DL after being exposed to a tutorial video on DL intubation. Group B performed intubation using VL, ProVu™ Single-Use Video Laryngoscope, after being exposed to a tutorial video outlining VL intubation. After watching the video, the students were not given feedback during their intubation attempts. Upon successful intubation using their respective devices, the groups switched; Group A participants then performed intubation using the VL after watching the VL tutorial, and Group B participants performed intubation using the DL after watching the DL tutorial. The number of attempts to successfully perform endotracheal intubation and the time for successful endotracheal intubation were recorded. A maximum of five attempts were allowed by each student, and those unable to attempt successfully within these attempts were designated as unsuccessful intubations. Participants then completed a post-questionnaire assessing their thoughts on training with VL compared to DL (Appendix 2).

Given the cross-over design, sample means and standard deviations were calculated for each method (VL, DL) based on the cross-over sequence. Means and standard deviations were used for time until intubation or failure, as well as the number of attempts. The proportion of students failing within five attempts was also summarized by sequence and method. We then tested for carry-over effects within sequences. Once it was confirmed that significant carry-over effects were not observed, an analysis of variance model was built to test for outcome differences between methods. A Chi-square test was also performed to determine whether there was evidence of imbalance in previous intubation experience across sequences.

## Results

Of the 93 participants, 15 reported no previous intubation experience. Among the participants reporting intubation experience, 69 reported a novice skill level, nine reported an intermediate skill level, and none reported a master skill level. Forty-nine students were assigned to Group A while 43 were assigned to Group B. We first assessed the carry-over effect and found no significant difference between groups for time to intubation (p = 0.0766), number of attempts (p = 0.1281), and proportion failing (p = 0.3080), as seen in Tables 1, 2.

**Table 1:** Means and standard deviations of outcomes among all participants VL: video laryngoscope, DL: direct laryngoscope

	Group A		Group B	
Outcome	Method = DL	Method = VL	Method = VL	Method = DL
Time to intubation (minutes)	2.37 ± 1.74	2.56 ± 1.63	1.26 ± 1.06	2.67 ± 1.92
Number of attempts	2.41 ± 1.59	2.39 ± 1.65	1.63 ± 1.14	2.41 ± 1.69
Proportion failing five attempts	16.32%	20.4%	6.80%	18.18%

**Table 2:** Tests for difference between methods among all participants VL: video laryngoscope, DL: direct laryngoscope

Outcomes	Mean difference between VL and DL	95% Confidence Interval	p-value
Time (minutes)	-0.61	(-0.99, -0.22)	0.0024
Attempts	-0.40	(-0.81, 0.01)	0.0582
Proportion failing	-3.60%	(-13.40%, 6.11%)	0.4603

**Figure 1:**
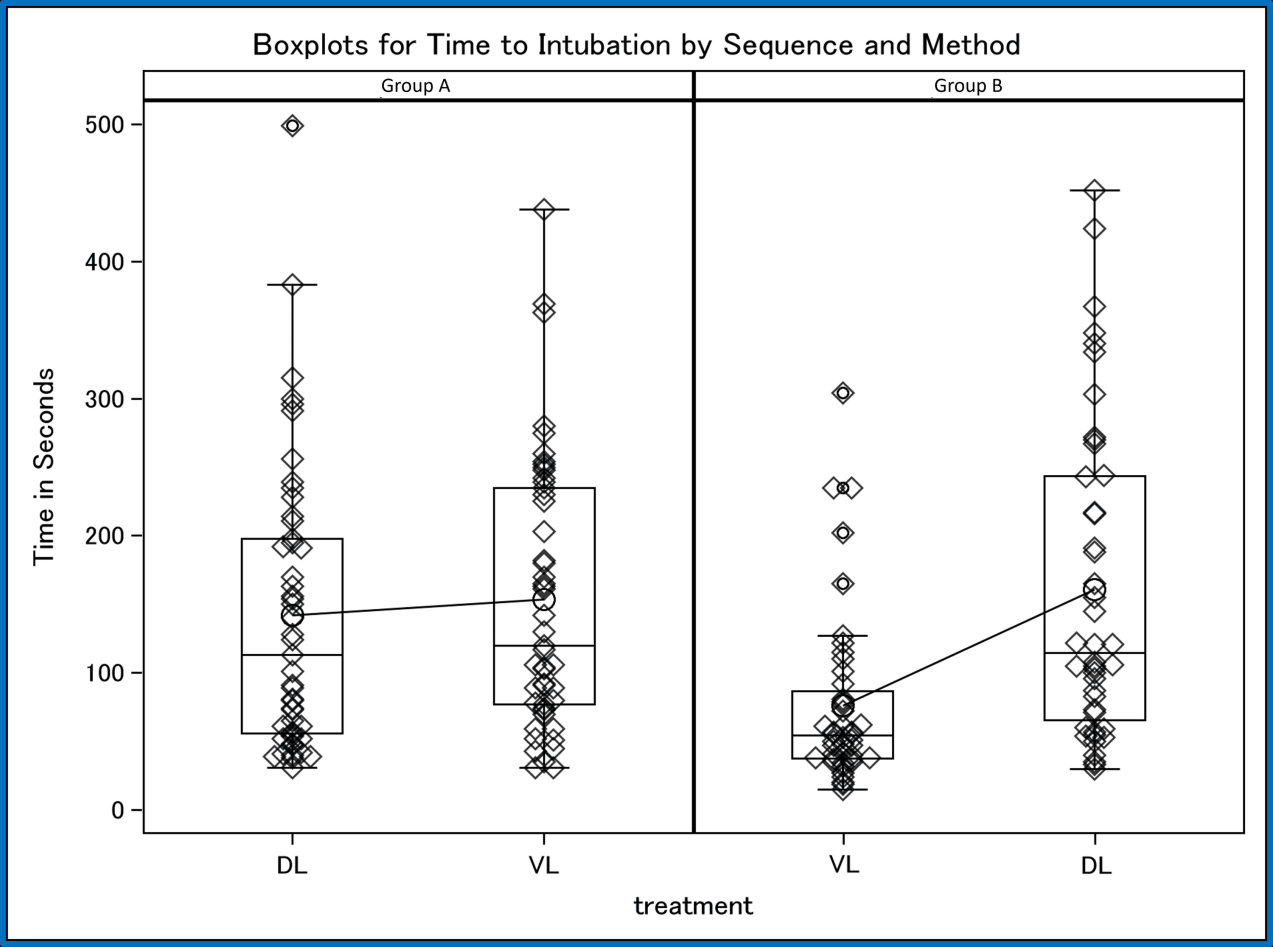
Boxplots for time to intubation by sequence and method. Sequence A shows Group A starting with DL then switching to VL. Sequence B shows Group B starting with VL then switching to DL. VL: video laryngoscope, DL: direct laryngoscope

We then analyzed for outcome differences by intubation device used among all participants and observed that VL produced a statistically significant (p = 0.0024) lower intubation time, on average 37 seconds faster (Figure 1). We also observed that VL produced fewer attempts, on average 0.40 attempts fewer, though this difference did not quite achieve statistical significance (p = 0.0582). Finally, we observed a slightly lower proportion of failure for VL, 3.6% fewer, though this was not statistically significant (p = 0.4603).

A Chi-square test was performed to assess whether there was potential imbalance in previous intubation experience among participants across sequences. The proportion reporting previous intubation experience in Group A was 77.55% and the proportion reporting previous intubation experience in Group B was 88.37% with a p-value of 0.1719, indicating no statistically significant difference between groups. Tables 3, 4 summarize the means and standard deviations of outcomes among participants without previous experience, whereas Tables 5, 6 report these statistics for participants with prior intubation experience.

**Table 3:** Means and standard deviations of outcomes in participants who had no previous experience VL: video laryngoscope, DL: direct laryngoscope

	Group A (N = 11)		Group B (N = 5)	
Outcome	Method = DL	Method = VL	Method = VL	Method = DL
Time to intubation (minutes)	2.17 ± 1.42	2.82 ± 1.75	0.69 ± 0.29	1.79 ± 2.25
Number of attempts	2.45 ± 1.75	2.55 ± 1.57	1.20 ± 0.45	1.80 ± 1.79
Proportion failing five attempts	18.18%	18.18%	0.00%	20.00%

**Table 4:** Tests for difference between methods among participants who had no previous intubation experience VL: video laryngoscope, DL: direct laryngoscope

Outcomes	Mean difference between VL and DL	95% Confidence Interval	p-value
Time (minutes)	-0.23	(-1.36, 0.90)	0.6724
Attempts	-0.25	(-1.53, 1.02)	0.6752
Proportion failing	-10.00%	(-35.87%, 15.87%)	0.4209

**Table 5:** Means and standard deviations of outcomes in participants who had previous intubation experience VL: video laryngoscope, DL: direct laryngoscope

	Group A (N = 38)		Group B (N = 38)	
Outcome	Method = DL	Method = VL	Method = VL	Method = DL
Time to intubation (minutes)	2.42 ± 1.84	2.49 ± 1.62	1.27 ± 1.02	2.81 ± 1.89
Number of attempts	2.39 ± 1.57	2.34 ± 1.70	1.61 ± 0.29	2.52 ± 1.69
Proportion failing five attempts	15.79%	21.05%	5.26%	18.42%

**Table 6:** Tests for difference between methods in participants who had previous intubation experience VL: video laryngoscope, DL: direct laryngoscope

Outcomes	Mean difference between VL and DL	95% Confidence Interval	p-value
Time (minutes)	-0.73	(-1.16, -0.32)	0.0008
Attempts	-0.49	(-0.92, -0.05)	0.0290
Proportion failing	-3.95%	(-14.65%, 6.76%)	0.4649

## Discussion

The goal of this study was to assess whether the VL would provide superior training outcomes for medical students when performing pediatric intubations compared to the DL. The post-questionnaire results revealed that 75% of students reported that the VL was highly effective in identifying landmarks for successful intubation. Additionally, 64% of students agreed or strongly agreed that the VL would serve as an effective teaching tool compared to the DL. This highlights the VL’s potential as a valuable resource in enhancing intubation training in medical education. Our quantitative data showed that students using the VL achieved a faster time to intubate compared to students using the DL. Although not statistically significant, the VL required fewer attempts on average, and its p-value indicated there may be a closer correlation present. These results suggest that the VL may offer valuable potential as a more efficient training tool for pediatric intubation. 

A prospective multi-institutional observational study was conducted using the National Emergency Airway Registry for Children (NEAR4KIDS) that focused on critically ill pediatric patients and their increased risk for adverse events resulting from intubation. The study found that the odds of patients experiencing low blood oxygen concentration increased with the increasing amount of tracheal intubation (TI) attempts. This study adds to current evidence that increasing the amount of TI attempts in critically ill children leads to an increase in adverse outcomes [4]. Although not statistically significant, a reduction in attempts seen in participants using the VL device warrants further investigation. The potential for reducing attempts in clinical practice by training with VL devices alongside DL devices should be further explored.

For example, one study evaluated the use of both direct and video laryngoscopy among inexperienced medical students and found that VL was more user-friendly and easier to learn. In addition, the initial attempts showed higher success rates and shorter intubation times in VL compared to DL [5]. Similarly, pulmonary and critical care fellows demonstrated a 34% higher first-attempt success rate with VL than with DL [6]. Another study showed that pediatric residents achieved significantly greater first-attempt success with VL compared with DL (88% vs. 66%) [7]. Across multiple stages of training, VL consistently demonstrates improved intubation success, underscoring its value as an effective teaching and performance tool for medical students.

Notably, early exposure to VL shortened intubation time following device crossover, suggesting that VL-acquired skills are transferable to DL technique [7]. In contrast, our study did not produce a significant carry-over effect among participants, which may be attributable to limited sample size. It is possible that with a larger cohort, prior VL training could yield reduced intubation times and fewer attempts when subsequently using DL.

Similarly, a video-enhanced educational curriculum was associated with faster intubation times and lower odds of requiring multiple attempts. A randomized controlled educational trial for pediatric residents at the Boston Children’s Hospital was performed to compare time to successful intubation between two groups. The control group was given the standard curriculum, including still images followed by simulation of an airway. The intervention group received a video-enhanced curriculum including deidentified intubation clips recorded using a videolaryngoscope which was then followed by simulation of an airway. The results showed that the median time to successful intubation in the intervention group was 18.5 seconds, whereas the control group had a median time to successful intubation of 22 seconds [8]. This study reinforces the idea that including video instructions can lead to better outcomes in endotracheal intubation simulations. Prior to beginning their attempts at endotracheal intubation, the subjects in our study viewed two short videos that demonstrated a step-by-step guide on both VL and DL intubation techniques, which may have influenced overall performance. In neonates, further evidence showed that VL use may reduce airway-related adverse events and overall decrease procedural harm when compared with DL [9]. Beyond technical performance, the VL enhances airway management by providing a shared real-time view of the airway, which facilitates team communication and supports collaborative problem-solving when anatomical or pathological challenges arise [10]. 

The majority of subjects in our study were at the “novice” level of experience, which limits the generalizability of the study to more experienced trainees. Another limitation of the study was the use of only a single VL device brand, which may restrict generalizability of our finding. Lastly, our assessment focused narrowly on time and attempts instead of accuracy of tube placement or airway trauma, which limits the real-world applicability.

## Conclusions

While many studies demonstrate the effectiveness of VL in improving intubation skills across different levels of medical training, there is limited evidence evaluating its impact when implemented during the early didactic years of medical school. The ability to view landmarks on the VL serves as an effective teaching tool that may enhance intubation training in pediatric simulation. The VL may improve students’ performance with the DL by familiarizing them with critical aspects they need to perform a successful intubation. Insufficient training in pediatric intubation, particularly in identifying anatomical landmarks and mastering correct techniques, may contribute to the potential implication of high rate of adverse effects in real-life pediatric intubation rates. This study contributes to the literature as there are limited studies showing the benefits of VL in a pediatric simulation for medical students. The VL has the potential to significantly enhance the students’ understanding of key anatomical landmarks and proper intubation techniques. 

## Appendices

Appendix 1: Pre-questionnaire

1.     Have you ever performed a knee aspiration? 

2.     If you answered yes to the above question, how would you rate your skill?

a.     Novice: practiced on a manikin during a training event

b.     Intermediate: you have 1-3 years of experience in the field where you performed this procedure occasionally

c.     Master: you have 3+ experience where you performed each technique regularly

3.     Have you ever placed a thoracostomy tube (chest tube)?

4.     If you answered yes to the above question, how would you rate your skill?

a.     Novice: practiced on a manikin during a training event

b.     Intermediate: you have 1-3 years of experience in the field where you performed this procedure occasionally

c.     Master: you have 3+ experience where you performed each technique regularly

5.     Have you ever intubated a patient? (adults, pediatrics or neonates)

6.     If you answered yes to the above question, how would you rate your skill?

a.     Novice: practiced on a manikin during a training event

b.     Intermediate: you have 1-3 years of experience in the field where you performed this procedure occasionally

c.     Master: you have 3+ experience where you performed each technique regularly

7.     Have you ever placed a central line?

8.     If you answered yes to the above question, how would you rate your skill?

a.     Novice: practiced on a manikin during a training event

b.     Intermediate: you have 1-3 years of experience in the field where you performed this procedure occasionally

c.     Master: you have 3+ experience where you performed each technique regularly

9.     Have you ever performed a cricothyrotomy?

10.  If you answered yes to the above question, how would you rate your skill?

a.     Novice: practiced on a manikin during a training event

b.     Intermediate: you have 1-3 years of experience in the field where you performed this procedure occasionally

c.     Master: you have 3+ experience where you performed each technique regularly

11.  Have you ever placed an intravenous line?

12.  If you answered yes to the above question, how would you rate your skill?

a.     Novice: practiced on a manikin during a training event

b.     Intermediate: you have 1-3 years of experience in the field where you performed this procedure occasionally

c.     Master: you have 3+ experience where you performed each technique regularly

Appendix 2: Post-questionnaire

1.     When compared to the direct laryngoscopy (DL) method, the video laryngoscopy (VL) was EASIER to perform endotracheal intubation on pediatric simulation.

a.     Strongly disagree, disagree, neutral, agree, strongly agree

2.     When compared to the DL method, I feel I would be more confident in performing a success endotracheal intubation on a pediatric simulation using the VL.

a.     Strongly disagree, disagree, neutral, agree, strongly agree

3.     When compared to the DL method, I found the VL to be effective tool to teach endotracheal intubation on a pediatric simulation.

a.     Strongly disagree, disagree, neutral, agree, strongly agree

4.     When compared to the DL, I found the VL to be an effective tool to identify anatomical landmarks for successful intubation.

a.     Strongly disagree, disagree, neutral, agree, strongly agree
